# Establishment of a Novel Primary Human Skeletal Myoblast Cellular Model for Chikungunya Virus Infection and Pathogenesis

**DOI:** 10.1038/srep21406

**Published:** 2016-02-19

**Authors:** Khairunnisa’ Mohamed Hussain, Regina Ching Hua Lee, Mary Mah-Lee Ng, Justin Jang Hann Chu

**Affiliations:** 1 Laboratory of Molecular RNA Virology and Antiviral Strategies, Department of Microbiology and Immunology, Yong Loo Lin School of Medicine, National University Health System, National University of Singapore, Singapore; 2 Department of Microbiology and Immunology, Yong Loo Lin School of Medicine, National University Health System, National University of Singapore, Singapore

## Abstract

Chikungunya virus (CHIKV) is a re-emerging arbovirus known to cause chronic myalgia and arthralgia and is now considered endemic in countries across Asia and Africa. The tissue tropism of CHIKV infection in humans remains, however, ill-defined. Due to the fact that myositis is commonly observed in most patients infected with CHIKV, we sought to develop a clinically relevant cellular model to better understand the pathogenesis of CHIKV infection. In this study, primary human skeletal muscle myoblasts (HSMM) were established as a novel human primary cell line that is highly permissive to CHIKV infection, with maximal amounts of infectious virions observed at 16 hours post infection. Genome-wide microarray profiling analyses were subsequently performed to identify and map genes that are differentially expressed upon CHIKV infection. Infection of HSMM cells with CHIKV resulted in altered expressions of host genes involved in skeletal- and muscular-associated disorders, innate immune responses, cellular growth and death, host metabolism and virus replication. Together, this study has shown the establishment of a clinically relevant primary human cell model that paves the way for the further analysis of host factors and their involvement in the various stages of CHIKV replication cycle and viral pathogenesis.

Chikungunya virus (CHIKV) is an arthropod-borne virus belonging to the genus *Alphavirus* within the family *Togaviridae*^1^. Since it was first reported present in the serum of a febrile individual from the Makonde Plateau in Tanzania, Africa in 1952^2^, CHIKV infections have mostly been documented as isolated cases in Africa and India^3^. Nevertheless, recent outbreaks have intensified efforts to understand biological events of this medically important human pathogen. In particular, the severe outbreak in the Indian Ocean island of La Réunion in 2005–2006 infected 38.2% of the population, resulting in 237 deaths^4^. Along with other widely recognised alphaviruses such as Sindbis (SINV), Semliki Forest (SFV), Ross River (RRV) viruses, CHIKV is responsible for high morbidity rates, accounting for millions of adverse, albeit nonfatal cases^5^. The re-emergence of CHIKV reiterates the potential threat that it poses to human health, and the need to investigate mechanisms involved in CHIKV biology.

Several studies have demonstrated susceptibility of various cell lines to CHIKV infection, with the rapid induction of apoptosis observed. Epithelial cells, endothelial cells, primary fibroblasts and monocyte-derived macrophages were found to be permissive to infection^6^. Skeletal muscle progenitor cells or satellite cells were also found to sustain CHIKV antigens^7^, possibly resulting in the recurrent myalgia experienced by infected individuals. Moreover, *in vivo* murine studies suggest fibroblasts as the primary cellular target for CHIKV infection^8^, confirming previous *in vitro* findings and accounting for CHIKV muscular and arthralgic tropism. Consistent with reports of neurological involvement, neurons and glial cells are also observed to be susceptible to CHIKV infection. In a non-human primate model, persistent infection of liver tissues, as well as significant levels of hepatocyte cell death implicates the involvement of hepatocytes in the disease^9^. Most recently, several mammalian and insect cell lines were assessed for CHIKV infection and host responses as a result of the infection^10^.

Nevertheless, the tissue tropism of CHIKV infection in humans remains to be fully defined, and a human cellular model that provides a true reflection of the infection is lacking. In this study, we identified and characterised human skeletal muscle myoblasts (HSMM) as a novel human primary cell line that is highly permissive to CHIKV infection. Furthermore, we performed genome-wide microarray profiling analyses of this cell line upon CHIKV infection to identify and map genes that are differentially expressed. Infection of HSMM cells with CHIKV viral particles resulted in altered expressions of host genes involved in several biological pathways. Subsequent quantitative real-time PCR assays on selected host genes confirmed the relevance of these genes during CHIKV infection. This work paves the way for further analysis of these host genes and their involvement in the various stages of CHIKV replication cycle.

## Results

### CHIKV infectability in HSMM

**Figure 1:**
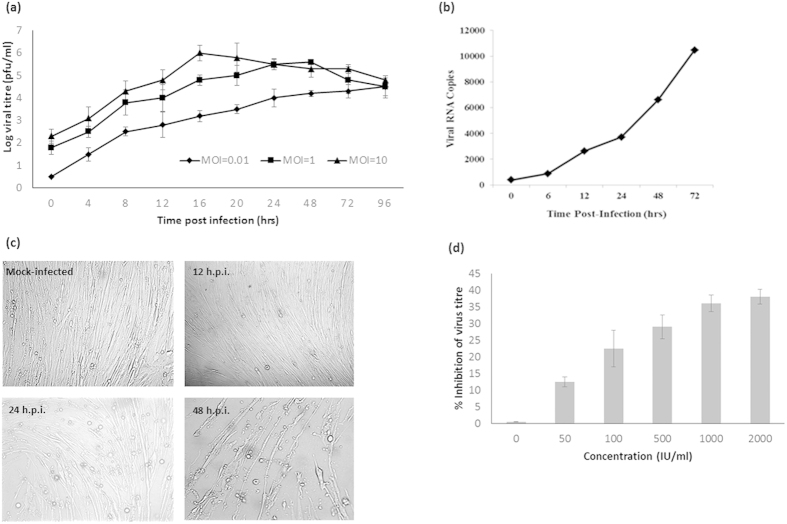
Susceptibility of HSMM to CHIKV infection. (**a**) Growth kinetics of CHIKV (an ECSA lineage, low passage Singapore strain 072008) on HSMM cells infected at different MOI of 0.1, 1 and 10. The plots shown are representative of three independent experiments. (**b**) qRT-PCR analysis of productive viral RNA copies of CHIKV infection on HSMM cells at MOI of 10. (**c**) Cytopathic effects observed upon CHIKV infection by 48 h.p.i. Mock-infected HSMM cells displayed typical fibroblastic features. (**d**) Pretreatment of HSMM cells with of type I interferon inhibited CHIKV infection in a dosage dependent manner.

Although several human cell lines have previously been shown to be permissive to CHIKV infection, these cell lines do not, however, address the cellular tropism observed during the infection in humans with most patients showing clinical manifestations of myositis. Primary human skeletal muscle myoblast (HSMM) cells were therefore utilized in this study to investigate the infective processes of CHIKV in its natural site of infection. Susceptibility of HSMM cells to CHIKV infection with an ECSA lineage, low passage Singapore strain 072008 via growth kinetics, was first established. As shown in Fig. 1a, the growth kinetics CHIKV strain Singapore 072008 was observed upon infection at the different MOI of 0.1, 1 and 10 on HSMM cells. HSMM cells infected with the MOI of 10 revealed peak virus production at 16 h.p.i. with approximately 10^6^ pfu/ml before subsequently plateauing at later infection time-points. In contrast, HSMM cells infected with lower MOI of 0.1 and 1 showed characteristics of multistep growth curve as the amount of infectious virus production increased steady with time post-infection. In addition, qRT-PCR analysis was carried out for HSMM cells infected with CHIKV strain Singapore 072008 at the MOI of 10 across the different time points. The amount of viral RNA was found to increase over time post-infection (Fig. 1b), with an approximately ten-fold increase over 24 hrs, and 27-fold after 72 h.p.i. of CHIKV infection. Morphological assessment of the HSMM cells upon CHIKV infection at MOI of 10 also revealed drastic cytopathic effects by 48 h.p.i., as compared to the fibroblastic nature of uninfected cells (Fig. 1c). Given the importance of type I interferons in the control of CHIKV infection^6^, the sensitivity of CHIKV infection of HSMM upon pretreatment of universal type I interferon was performed. Pretreatment of HSMM cells with universal human type 1 interferon inhibited the replication and production of infectious CHIKV in a dosage dependent manner (Fig. 1d) hence further supporting the relevance of this primary cellular model for CHIKV infection.

**Figure 2:**
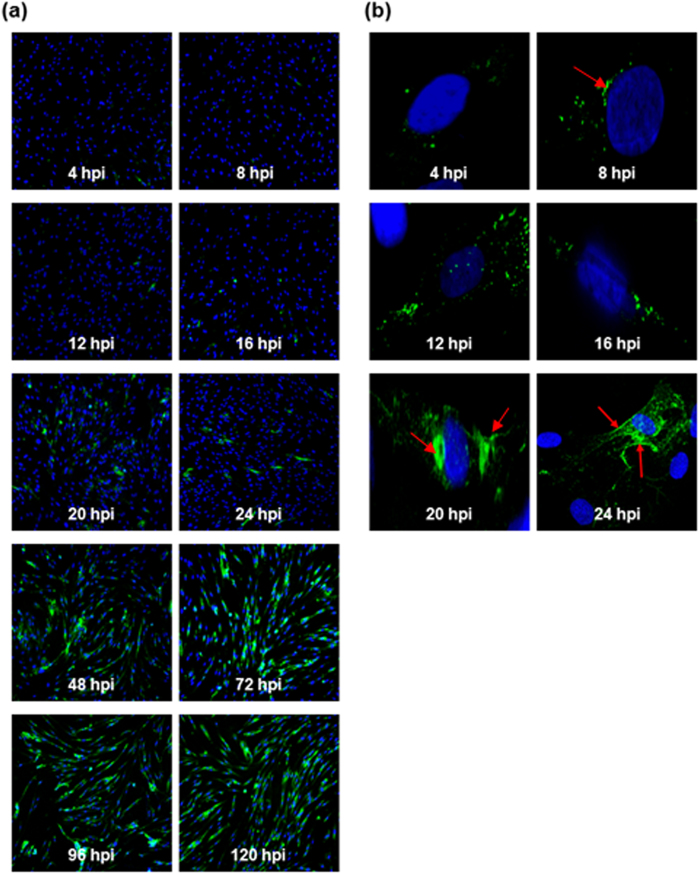
Immunofluorescence assay depicting CHIKV replication in HSMM cells (**a**) Production of CHIKV envelope protein (FITC) could be observed by 12 h.p.i., with almost complete infection of all HSMM cells observed by 72 h.p.i. (100x magnification). (**b**) At higher magnification, CHIKV viral antigens (arrows) are observed at the perinuclear region as early as 8 h.p.i. and subsequently localized to the cellular periphery by 24 h.p.i. (1000x magnification; arrows). Cell nuclei are counterstained with DAPI.

CHIKV (strain Singapore 072008) replication in HSMM cells was also analysed by performing an immunofluorescence assay. The production of the FITC-stained CHIKV envelope protein could be observed by 12 h.p.i. (Fig. 2a), with almost-complete infection rate by 72 h.p.i. In the early stages of infection, the viral E2 antigen could be observed within the perinuclear region of the cell (Fig. 2b). As infection progressed, the viral proteins (arrows) were observed to be accumulating within the cytoplasm and towards the plasma membrane. It is known that the viral envelope proteins are synthesized at the rough ER and transported to the plasma membrane for virus assembly and budding to release the infectious virus particles.

### CHIKV undergoes dual mode of egression from host cells

**Figure 3:**
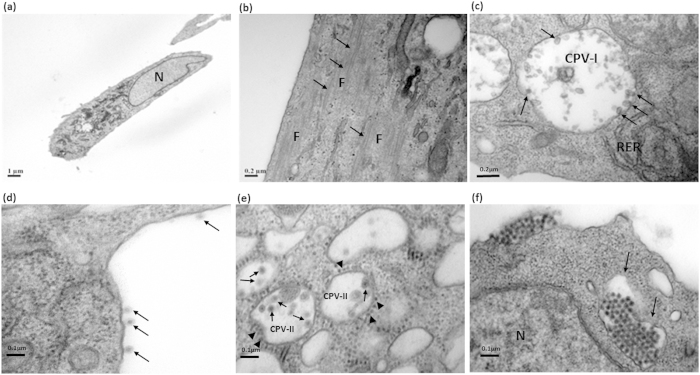
Transmission electron microscopy analysis of CHIKV infection in HSMM cells. (**a,b**) Mock-infected HSMM cells displayed normal fibroblastic features and this morphology remained similar across the time points till 96 h. (**c**) Formation of CPV-Is are observed at 6 h.p.i. and was followed by proliferation of rough ER and spherules within the CPV-I are indicated by the arrows. (**d**) Virus particles budding (arrows) at the plasma membrane can be observed at the early stage of infection (8 h.p.i.). (**e**) Numberous viral nucleocapsids (arrowheads) were subsequently observed to be budding into CPV-IIs to form mature virions (arrows) by 24 h.p.i. The bags of mature viruses within CPV-IIs are release at the plasma membrane at the late stage of infection. (**f**) Massive exocytosis at later infective stages were observed, with exocytic vesicles (arrows) containing mature virions. N denotes nucleus; F denotes: myofilaments; RER denotes rough endoplasmic reticulum. The scale bars are indicated in the figures.

Ultrastructurally, normal uninfected cells displayed long fibroblastic nature of cell morphology (Fig. 3a), with numerous actin/myosin filaments present (Fig. 3b).Upon CHIKV infection (strain Singapore 072008), virus-induced structures such as replication complexes could be observed. During early infective stages of between 4 to 8 h.p.i., spherules (arrows) - postulated to form the site of viral transcription^11^ - were observed within cytopathic vesicles-I (CPV-I) (Fig. 3c). The CPVs were closely associated with rough endoplasmic reticulum, which has been postulated to be the site of viral translation^12^. Virus particles are observed to be budding (arrows) at the plasma membrane during the early infection phase. The presence of viral envelope that enclosed the nucleocapsid is obvious from the budding virus particles at the plasma membrane (Fig. 3d). CPV-IIs were formed in later infective stages of between 24 to 48 h.p.i., with viral nucleocapsids (arrowheads) aligning themselves along the cytoplasmic vesicles prior to budding to form mature virions (arrows) within the CPV-IIs (Fig. 3e). The exocytotic vesicles (arrows) that originate from CPV-IIs containing numerous virions were subsequently transported to the plasma membrane to release the virus particles by the process of exocytosis (Fig. 3f). The significance of the dual mode of CHIKV egression, that is budding during early infective phase and exocytosis during late infective phase remains to be unravel, although it could provide an interesting clue as to the pathogenesis of CHIKV infection. In the event whereby the host mounts an early immune response against CHIKV infection, releasing inhibitory host factors to block viral exit and subsequent replication, the virus may then adopt an alternative mechanistic mode to counter host responses and enable mature virions to be released in huge numbers.

### CHIKV induces apoptosis in host cells

**Figure 4:**
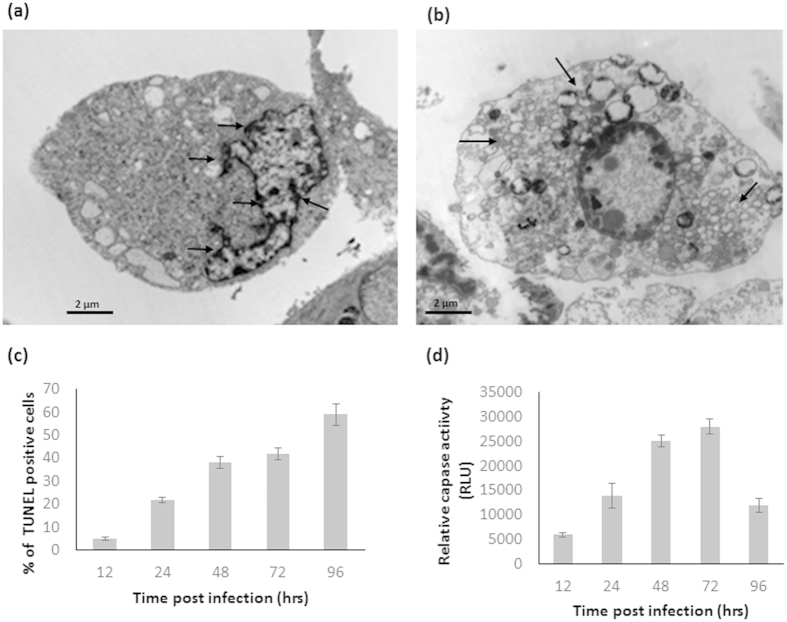
Ultrastructural analysis of the CHIKV-infected HSMM cells revealed (**a,b**) massive vacuolation and nucleolar degradation. (**c**) TUNEL positive HSMM cells increased in number with the progression of CHIKV infection. (**d**) Caspase 3 was activated by CHIKV-infected HSMM cells and peak caspase 3 activities was observed at 72 h.p.i.

Previous studies have shown that CHIKV can induce apoptosis in infected cells^13^,^14^. In order to determine whether CHIKV induces apoptosis in the infected HSMM cells, TEM analysis were also performed at late infection time-points (72 and 96 h.p.i.). Intense nuclear condensation (arrows, Fig. 4a) and widespread vacuolation (arrowheads, Fig. 4b), classical features of cellular apoptosis, were observed by 72 and 96 h.p.i. A TUNEL assay to detect *in situ* cell death further illustrated the apoptotic action induced upon CHIKV infection of HSMM cells, whereby the number of apoptotic cells increased as infection progressed from 24 to 96 h.p.i. (Fig. 4c). In addition, we analyzed caspase 3 activities in CHIKV strain Singapore 072008-infected HSMM cells and the mock-infected control. As shown in Fig. 4d, caspase 3 activities in HSMM cells infected with CHIKV increased substantially from 24 h.p.i. and peak at 72 h.p.i. These data indicate that CHIKV-induced apoptosis in HSMM cells is associated with the activation of caspase 3 associated pathway.

### Susceptibility of HSMM cells to different lineages and strains of CHIKV infection

**Figure 5:**
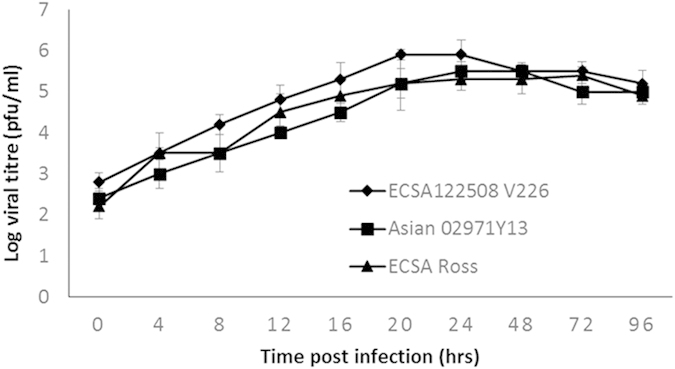
Susceptibility of HSMM cells to different lineages and strains of CHIKV infection. HSMM cells are infected with low passages of the ECSA lineage of CHIKV (Ross strain) and the CHIKV strain Singapore 1225Y08 which carries the alanine to valine mutation at position 226 (A226V) of the CHIKV E1 protein as well as the Asian lineage (Singapore 02971Y13) to assess for their ability to infect HSMM. HSMM cells are highly susceptible to infection by all the three chikungunya viruses. The plots shown are representative of three independent experiments.

The permissiveness of HSMM cells to different CHIKV from different lineages and strains were further assessed. The ECSA lineage of CHIKV (Ross strain) and the Asian lineage (Singapore 02971Y13) were assessed for their ability to infect HSMM. In addition, the CHIKV strain Singapore 1225Y08 which carries the alanine to valine mutation at position 226 (A226V) of the CHIKV E1 protein was also assessed for its ability to infect and replicate in HSMM cells. The A226V mutation was detected during the latter part of the outbreak in La Réunion and resulted in a change in mosquito species vector from *Aedes aegypti* to *Aedes albopictus*. As shown in Fig. 5, all the three viruses can effectively infect and replicate in HSMM cells. It was also noted that CHIKV strain Singapore 1225Y08 produce a slightly higher virus tier in HSMM cells than the CHIKV strain Ross and Singapore 02971Y13. Together, these findings presented so far strongly suggest that HSMM is a susceptible primary cellular model for CHIKV infection studies.

### CHIKV infection regulates HSMM cell gene expression

In order to better understand the pathogenesis of CHIKV infection, the effect of CHIKV infection on host gene expression was subsequently investigated by performing human genome-wide microarray profiling across four different time-points of CHIKV infection (using the CHIKV strain Singapore 072008) in HSMM cells – 6, 12, 24 and 48 h.p.i. Total RNA extracted from cells was hybridized to Illumina HumanHT-12 v4 Expression BeadChips for gene expression analysis.

**Figure 6:**
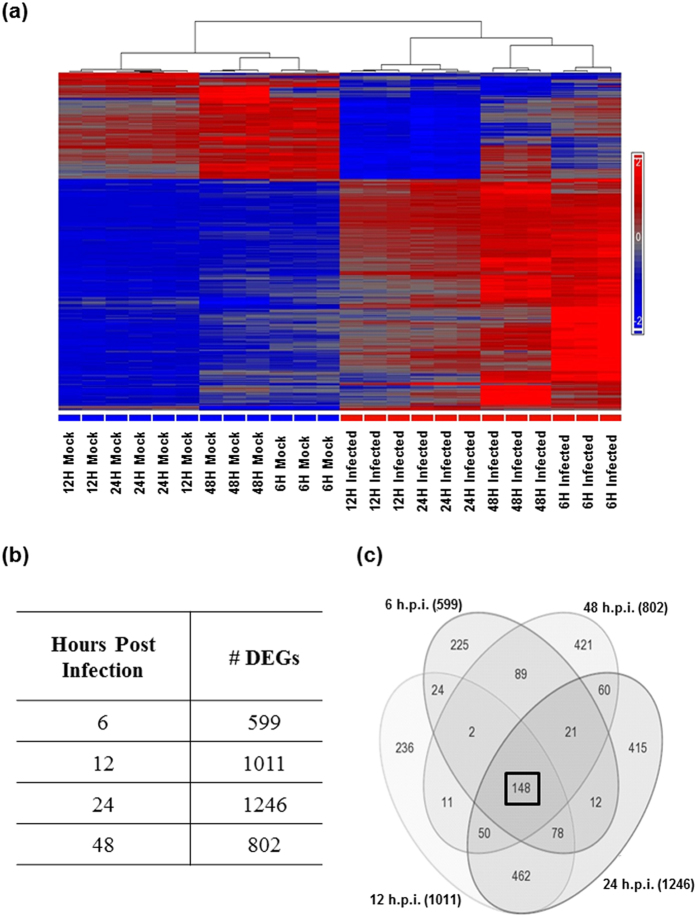
Genomic profiling and comparison analyses of CHIKV infection on HSMM cells (**a**) Heatmap displaying differential regulation in gene expression between CHIKV-infected and mock samples (**b**) Table with numbers of DEGs at various CHIKV infection time-points. (**c**) Venn diagram with 148 genes in common across all time-points (boxed).

As shown in Fig. 6a, a heatmap generated indicated distinct separation between mock and CHIKV-infected triplicate samples, regardless of infection time-points. Sample grouping was initially assessed with the use of the sources of variation and principal component analysis (PCA) plots, which further confirmed that the differences in gene regulation were indeed a result of CHIKV infection on HSMM cells (Supplementary Figs S1a and S1b). Throughout the microarray analyses, differential regulation in gene expression is defined as absolute fold-changes of more than 2, denoting biological significance, as well as *p* values of less than 0.05, denoting statistical significance. It was observed that while majority of genes were expectedly biologically and/or statistically insignificant, there were a good proportion of genes that fell within the criteria set for significance and were hence good candidates for further analyses (Supplementary Fig. S1c).

### Gene ontology analysis

The total number of differentially expressed genes (DEGs) between CHIKV-infected and mock-infected samples at each time-point is shown in Fig. 6b. A total of 148 genes – 143 up-regulated and 5 down-regulated across all four time-points – were subsequently selected for further analyses into the various pathways that may have been involved in causing these differences during CHIKV infection (Fig. 6c).

**Figure 7:**
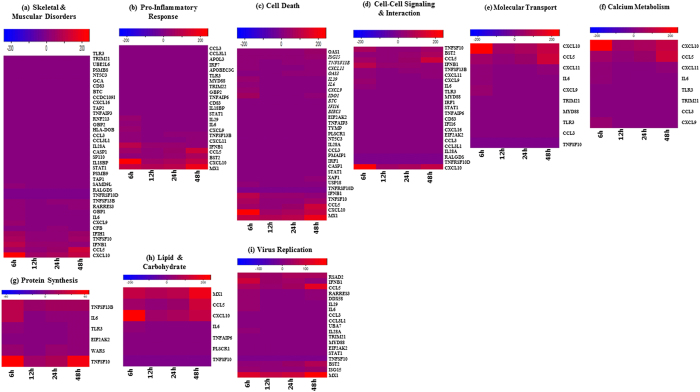
Heatmaps displaying various biological pathways of interest. Host genes with altered expression levels during CHIKV infection were involved in a range of biological processes, with nine functional pathways selected.

Host genes with altered expression levels during CHIKV infection were involved in a range of biological processes, and a total of nine functional pathways were subsequently selected (Fig. 7). Genes chosen for further investigation included those involved in pathways regulating host defense (e.g. inflammation, cell death), host metabolism (e.g. lipid and carbohydrate metabolism, protein synthesis) and virus replication. Many of the host genes significantly regulated by infection were also involved in processes such as vision, taste and embryogenesis. These processes were not considered with relevant importance to the study and were therefore excluded from the analysis.

### CHIKV infection alters the expression of host genes involved in skeletal and muscular disorders

As determined by IPA, skeletal and muscular disorders, such as arthritis and rheumatoid arthritis, were identified as the disease group most closely associated with CHIKV infection. This is hardly surprising as infection by CHIKV often results in persistent myalgia and polyarthralgia in infected patients. The expression profiles for 38 skeletal-muscular associated genes regulated by CHIKV infection are represented by heat map diagrams in Fig. 7a and the complete list of differentially regulated genes across the different time points p.i. are provided in Supplementary Fig. S2.

Among those differentially expressed genes in this group are GBP1, STAT1 and CXCL10, which have all been shown to be involved in muscle and bone metabolism^15^. Additionally, GBP1 was previously found to be associated with fibromyalgia^16^, while an increased expression of TAP1 was observed in juvenile dermatomyositis^17^.

### CHIKV infection alters the expression of genes involved in host defense mechanisms

Many of the genes regulated following CHIKV infection were involved in host defence mechanisms in order to protect the cell against the infection and ensure survival. The expression profiles for the cellular defence genes regulated by CHIKV infection are represented by heat map diagrams in Fig. 7.

Increases in expression levels in 23 genes associated with innate immune responses were observed upon CHIKV infection (Fig. 7b) including those involved in type I and II interferon responses (e.g. IRF1, IRF9, and MX1 genes), type III interferon responses (e.g. interleukin-29 [IL-29]) and the production of proinflammatory chemokines and cytokines (e.g. IL-6, GBP2, CCL3 and -5, and CXCL9, -10 and -11 genes). Increased expression of genes encoding negative regulators of the interferon response was also observed (e.g. IL18BP gene).

The expression of 31 genes controlling apoptosis and cell cycle arrest was significantly regulated by infection (Fig. 7c). Transcriptional changes that promoted apoptosis and cell cycle arrest were observed. These included an increase in the expression of genes that induce apoptosis, such as those involved in p53 and TGFβ signaling (e.g. IFI16 gene), an increase in the expression of pro-apoptotic genes (e.g. PMAIP1, CASP1, TNFSF10 and XAF1 genes) and a decrease in the expression of anti-apoptotic genes (e.g. TNFRSF10D gene). At the same time, increased expression levels were also observed in genes promoting inhibition of the apoptotic machinery (e.g. BIRC3 and TNFAIP3 genes) as well as genes promoting cellular survival and proliferation (e.g. TNFSF13B and PLSCR1 genes), demonstrating that conflicting transcriptional changes occurred following CHIKV infection.

### CHIKV infection alters the expression of genes involved in cellular functions

Genes involved in typical cellular functions were differentially expressed in spite of CHIKV infection to ensure continuous survival, including those associated with cell-cell signalling and interaction, molecular transport & shuttling and host metabolism. The expression profiles for the cellular function genes regulated by CHIKV infection are represented by heat map diagrams in Fig. 7.

Significant increases in the expression levels of 23 cell-cell signalling related genes were observed (Fig. 7d). These genes included those involved in the IL-1 and TLR signalling pathways such as TLR3 and MYD88, while EIF2AK2 regulates various other signalling pathways such as p38 MAP kinase, NFκB and insulin pathways. Conversely, RALGDS, known to be an effector of Ras-related GTPases that participate in signalling for a variety of cellular processes, was found to be down-regulated upon CHIKV infection.

Ten genes mediating molecular transport and shuttling in host cells were also found to exhibit increased expression levels following CHIKV infection (Fig. 7e). For instance, MYD88 has been shown to activate IRF1 expression resulting in its rapid migration into the nucleus. Other genes associated with molecular transport and shuttling include TRIM21, CCL5, TLR3 and CXCL11.

Several genes differentially expressed upon CHIKV infection were involved in host metabolic processes in order to ensure continual survival of the cell. A total of eight highly-regulated genes, which are more known for their inflammatory properties, are involved in calcium metabolism (Fig. 7f). These genes include CXCL11, IL6, TLR3, TRIM21, CCL3 and CXCL9. In particular, CXCL10 was found to be upregulated by approximately 100-fold while CCL5 expression was increased about 55-fold following CHIKV infection.

Increases in expression levels in six genes associated with protein synthesis were also observed upon CHIKV infection (Fig. 7g). For instance, expression of EIF2AK2, known to inhibit viral replication via inhibition of translation resulting in shutdown of both cellular and viral protein synthesis, was found to be enhanced by three-fold following CHIKV infection. Thought to be part of a pioneering group of proteins in evolution due to its central role in linking role in amino acids with nucleotide triplets contained in tRNAs, WARS was similarly observed to be highly expressed by five-fold upon CHIKV infection.

Significant increases in the expression levels of seven lipid and carbohydrate metabolism-related genes were observed (Fig. 7h), including TNFAIP6 and PLSCR1. The former, known to be involved in extracellular matrix stability and cell migration via its hyaluronan-binding domain, was observed to increase by approximately five-fold during CHIKV infection. Interestingly, enhanced levels of TNFAIP6 have previously been found in patients with osteoarthritis and rheumatoid arthritis^18^,^19^,^20^, thus further substantiating the CHIKV-induced levels observed in the microarray data. Meanwhile, known to mediate transbilayer phospholipid migration resulting in loss of phospholipid asymmetry in the plasma membrane, PLSCR1 was found to be enhanced by 4.5-fold upon CHIKV infection.

### CHIKV infection alters the expression of genes involved in virus replication

As determined by IPA, 19 genes associated with viral replication were found to be regulated by CHIKV infection and are represented by Fig. 7i. Among those highly expressed genes in this group include RARRES3, DDX58, BST2, MYD88 and MX1. In particular, RSAD2 or viperin was found to be largely enhanced by 50-fold upon CHIKV infection. Known to inhibit a wide range of DNA and RNA viruses, such as human cytomegalovirus (HCMV)^21^, hepatitis C virus (HCV)^22^, influenza A virus^23^, SINV^24^, West Nile virus (WNV) and dengue virus (DENV)^25^, RSAD2 has even been previously found to restrict CHIKV replication and pathology^26^, thus strengthening the findings in this study.

### Validation of microarray expression data

**Figure 8:**
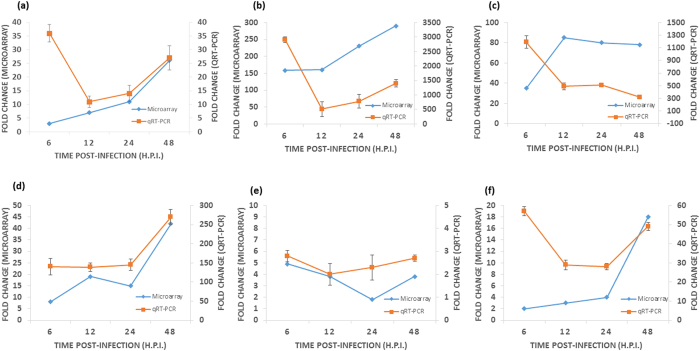
Comparison between microarray genomic profiling data and qRT-PCR validation data. Six top-hit genes [(**a**) RARRES3, (**b**) CXCL10, (**c**) IFNB1, (**d**) OAS1, (**e**) MYD88 and (**f**) RTP4] were randomly selected and exhibited enhanced mRNA levels upon CHIKV infection across all infection time-points. Three independent experiments have been carried out for the qRT-PCR.

In order to validate the microarray findings, six genes from the list of positive screening hits were randomly selected for qRT-PCR validation and subsequent comparison against the microarray data. As shown in Fig. 8, in line with the microarray results, all selected genes exhibited enhanced mRNA levels upon CHIKV infection across all infection time-points, albeit with varying degrees of fold changes relative to mock-infected samples and β-actin as the normalising housekeeping control. The square-marked graphs depict microarray data while qRT-PCR results are represented by the diamond-marked graphs. Both sets of results were taken from triplicate independent samples.

With the exception of the 6 hr infection time-point, the differential expression levels of RARRES3 across all infection time-points were showing similar trend for both the microarray and qRT-PCR data, with approximately 11-fold and 12-fold up-regulation at 12 h.p.i., respectively (Fig. 8a). Conversely, as shown in Fig. 8b, although microarray expression levels of CXCL10 only reached to approximately 240-fold upregulation, qRT-PCR validation levels were immensely enhanced to more than 3000-fold upon CHIKV infection. The contrasting difference in the fold changes between the microarray and qRT-PCR assays could be attributed to the sensitivity of the assay especially for qRT-PCR which is extremely sensitive.

In addition, it can be seen that microarray expression levels of IFNβ1 showed a similar downward trend from 12 h.p.i. to 48 h.p.i., albeit at much higher levels for the qRT-PCR data (Fig. 8c), OAS1 and MYD88 exhibited highly similar upward trend of upregulated expression levels for both data sets across all infection time-points, although at much enhanced levels for the qRT-PCR data (Figs 8d,8e, respectively). Finally, the upward trend of upregulated expression levels of RTP4 was observed in both sets of data from 12 h.p.i. to 48 h.p.i., with similar expression values seen at both 12 h.p.i. and 24 h.p.i. time-points (Fig. 8f).

## Discussion

Although several human cell lines have previously been shown to be permissive to CHIKV infection, these cell lines do not, however, address the tissue tropism observed during the infection in humans. Ozden and colleagues^7^ has previously described the involvement of skeletal muscle progenitor cells or muscle satellite cells to sustain CHIKV antigens but not in differentiated myotubes. Furthermore, *in vivo* studies by Rohatgi and colleagues^29^ revealed the role of muscle connective tissue fibroblasts and myofibers which could impact the severity of CHIKV infection in murine model. We have also previously shown the susceptibility a human rhabdomyosarcoma cell line (SJCRH30) to CHIKV infection^30^. However, the role and involvement of human myoblast in the pathogenesis of CHIKV remains elusive. To this end, HSMM cell was identified and characterised as a novel human primary skeletal myoblast cell line that is highly permissive to CHIKV infection. Our data has also revealed that the different lineages and strains of CHIKV can effectively infect HSMM and produces infectious virus progeny with high virus titre. Morphological assessment of the cells during CHIKV infection showed that cytopathic effects can be observed by as early as 12 h.p.i., with almost complete destruction of the cell monolayer seen by 48 h.p.i. CHIKV growth kinetics performed on HSMM cells also showed peak viral titres at 16 h.p.i. While these findings are consistent with various research done previously, the peak titres at a much earlier infection time-point suggest the high permissibility of HSMM cells to CHIKV infection.

In order to further understand the pathogenesis of CHIKV infection, the effect of CHIKV infection on host gene expression was analysed by performing human genomic microarray profiling. The Illumina HumanHT-12 v4 Expression BeadChip used targets more than 47,000 probes across the entire human transcriptome. Although several CHIKV-based microarray studies have been conducted recently^31^,^32^, no genome-wide analyses have been performed. In the case of Nakaya and colleagues^32^, only a consensus CHIKV arthritis gene expression signature in a mouse model was used while Lee and co-workers^31^ conducted microarray profiling targeting less than 19,000 transcripts across the mosquito genome. This study demonstrates, for the first time, genome-wide profiling across the human transcriptome in a primary human myoblast cell model for CHIKV. A total of 148 genes was differentially regulated with 143 host genes being up-regulated and 5 down-regulated host genes across all four infection time-points. The highest number of differentially regulated host genes was observed at 24 h.p.i., which coincides with the peak of CHIKV infection in HSMM cells, thus suggesting that a maximal number of host genes were regulated during this period, either due to host defence or as a means of providing aid to the virus. A large numbers of these differentially regulated host genes are clustered under the similar functional pathways that contribution to skeletal and muscle disorder. In addition, the validation of the differentially regulated host genes via qRT-PCR supported the importance of these host genes in mediating the skeletal- and muscular-associated disorders, innate immune responses, cellular death, cellular metabolism, hence possibly leading to virus replication and pathogenesis of CHIKV infection. It was interesting to note that there were conflicting transcriptional changes in the expression of pro-apoptotic genes and anti-apoptotic genes that were observed at the same time point. During virus infection, it is a struggle of the host cells to maintain cellular haemostasis and regulation of programmed cell death pathways. The host cell may induce apoptotic pathway as an innate immune response to get rid of the virus infection. In contrast, the virus may regulate the host genes towards pro-survival function to ensure the survival of host cells for its replication. Recently, it has been documented that the regulation of autophagy by CHIKV infection to promote pro-survival of cells upon infection^33^.

In conclusion, novel findings have been established throughout the study in relation to the interactions between CHIKV and its host through the development of a primary human myoblast cellular model for CHIKV infection. Further studies are crucial to understanding the mechanisms of CHIKV pathogenesis in this primary cell model. It is only through deep and thorough understanding of the pathogenesis of this virus infection that we will be able to develop effective, targeted therapeutics.

## Methods

### Cells and Viruses

Human primary skeletal muscle myoblasts (HSMM) (CC-2580) (Clonetics, Lonza Group Ltd., Switzerland) were maintained in Skeletal Muscle Myoblast Cell Growth Medium (SkGM-2) supplemented with 10% fetal bovine serum (FBS), dexamethasone, GA-1000, rhEGF and L-glutamine (Clonetics) at 37 °C with 5% CO_2_. The HSMM cells that were purchased from Lonza were isolated from anonymized normal donors. HSMM are isolated from the upper arm or leg muscle tissue of the healthy donors. All experiments with HSMM were performed in accordance with the approved guidelines given by the National University of Singapore Institutional Review Board. Baby hamster kidney (BHK-21) cells (American Type Culture Collection, ATCC CCL-10) were maintained in Roswell Park Memorial Institute (RPMI-1640) (Sigma-Aldrich Corp., U.S.A.) growth medium supplemented with 10% FBS (Hyclone, U.K.) and sodium bicarbonate (Merck).

Low passage CHIKV ECSA lineage strains [Singapore 072008, Singapore 1225Y08 (NCBI Accession: FJ445502.2) and Ross] as well as the Asian lineage (Singapore 02971Y13)] were propagated in C6/36 mosquito cells. The CHIKV strain Singapore 072008 was obtained from Dr Raymond Lin (National Public Health Laboratory, Ministry of Health, Singapore). The CHIKV strains Singapore 1225Y08 and Singapore 02971Y13 were kind gift from Dr Ng Lee Ching (Environmental Health Insitute, National Environmental Agency, Singapore). The CHIKV strain Ross was kindly provided by Dr Ooi Eng Eong (Duke-NUS, Singapore). All experiments involving the use of the CHIKV strain were performed in Biosafety Level 2 facilities.

### Virus titration and growth kinetics studies

Viral plaque assays were performed on BHK-21 cells to quantitate infectious virus titers, calculated following the method of Dulbecco^27^. Briefly, BHK-21 cell monolayers (7 × 10^4^ cells/well) in 24-well tissue-culture trays (Cellstar, Greiner Bio-One, Germany) were inoculated with 100 μL 10-fold serial dilutions of each sample and incubated for 2 hrs at 37 °C prior to the addition of overlay medium and incubation for 3 days. The presence of plaques was subsequently observed by crystal violet staining.

HSMM cell monolayers grown overnight in 24-well tissue culture trays (1 × 10^5^ cells/well) were infected with CHIKV strain Singapore 072008 at a multiplicity of infection (MOI) of 0.1, 1 and 10 for 2 hrs at 37 °C. Inoculated cells were then washed three times with PBS and overlaid with 1 mL maintenance medium (SkGM-2 supplemented with 2% FBS) per well. Cells and supernatants were collected at regular intervals until 96 hrs post-infection, and with the first time point (time 0) collected immediately after the addition of the maintenance medium to account for excess non-specific virus binding. Infectious virus titers of the supernatants collected were determined by viral plaque assay as previously mentioned. Viral RNA was also extracted (Qiamp viral RNA kit; Qiagen, Germany) from viral supernatants obtained from parallel experiments conducted, and titrated by quantitative real-time reverse transcriptase PCR (qRT-PCR). In addition, HSMM cells were infected with the different CHIKV strains [ECSA lineage: Singapore 1225Y08 (NCBI Accession: FJ445502.2) and Ross and Asian lineage (Singapore 02971Y13)] at MOI of 10 and supernatants were harvested for plaque assays at regular intervals until 96 hrs post-infection. For the interferon type I treatment assay, HSMM cells were treated with universal interferon type 1 (PBL Assay Science) at 50 to 200IU/ml for 2 hrs before infection with CHIKV strain Singapore 072008 at a MOI of 10. The cell culture supernatants were harvested at 24 h.p.i. for viral plaque assays.

### Indirect Immunofluorescence Microscopy

HSMM cell monolayers grown overnight on coverslips till 75% confluency were infected with CHIKV strain Singapore 072008 at an MOI of 10 for 2 hrs at 37 °C. Infected cells were fixed in ice-cold absolute methanol and washed three times with cold PBS. The cells were then subjected to immunofluorescence staining using primary customised rabbit polyclonal antibodies targeted against CHIKV E2 protein (ProSci Inc., USA) at a 1:100 dilution, followed by secondary antibodies conjugated to fluorescein isothiocyanate (FITC; Invitrogen) at a 1:1000 dilution. Cell nuclei were counter-stained with 4′, 6′-diamidino-2-phenylindole (DAPI; 100 nM) fluorescent dye (Invitrogen) at a 1:250,000 dilution. The specimens were then viewed with Olympus IX81 motorized inverted epifluorescence microscope (Olympus, Japan) with appropriate excitation and emission wavelengths for FITC (490 nm and 525 nm respectively), and DAPI (350 nm and 470 nm respectively) at 100x and 1000x magnification.

### Transmission Electron Microscopy

To track CHIKV (strain Singapore 072008) infectious processes into HSMM cells at various time points p.i, CHIKV-infected cells were fixed with 2.5% glutaraldehyde (Agar Scientific, U.K.) at 4 °C for 20 mins, followed by scraping of the cells and further subjecting them to fixation at 4 °C overnight. The following day, cells were centrifuged and the pellets were washed with PBS and deionized water. Cell pellets were post-fixed with 1% osmium tetroxide (Ted Pella, Redding, USA) and 1% potassium ferro-cyanide for 2 hrs, followed by dehydration in an ascending graded series of ethanol and acetone, i.e. 25%, 50%, 75%, 95% and 100% for 10 mins at each concentration. Cells were then infiltrated with resins by passing them through three changes of mixture, comprised of a combination of acetone, ethanol and araldite. The following day, cells were infiltrated with four changes of absolute embedding media with 1 hr incubation at room temperature, 40 °C, 45 °C and 50 °C. After the last spin, cell pellets were resuspended in 100–200 μl araldite. Mixture was embedded using the BEEN capsule (size 3) and was incubated at 60 °C for 24 hrs to allow polymerization. The samples were trimmed with an ultramicrotome (Reichert-Jung, U.S.A.) and the sections were stained with 2% uranyl acetate and fixed with lead citrate. The stained sections were viewed under the transmission electron microscope Philip EM 208 and images were captured digitally with a dual view digital camera (Gatan, U.S.A.).

### TUNEL apoptotic and Caspase 3 detection assays

A terminal deoxynucleotidyl transferase (TdT) dUTP nick-end labelling (TUNEL) assay was performed using the *In Situ* Cell Death Detection Kit, Fluorescein (Roche, U.S.A.), according to manufacturer’s instructions. Briefly, HSMM cell monolayers grown overnight on coverslips till 75% confluency were infected with CHIKV strain Singapore 072008 at an MOI of 10 for 2 hrs at 37 °C. Infected cells were subsequently fixed with 4% paraformaldehyde/PBS and permeabilised with 0.1% Triton-X/0.1% sodium citrate solution at daily time-points. Negative (without TdT) and positive (treated with 1 μM staurosporine for 2 hrs and harvested at 8 hrs post-infection [h.p.i.]) labeling controls were included, along with mock-infected controls. Samples were then subjected to labeling with the TUNEL reaction mixture prior to imaging analysis. Cell nuclei were counter-stained with DAPI. HSMM cells were infected with CHIKV strain Singapore 072008 at MOI of 10. At selected time intervals p.i., CHIKV-infected cells were processed in accordance with manufacturer’s instructions (Promega Caspase-Glo® 3/7 Assay kit) to determine caspase-3 activity. Luminescence of each sample well was analyzed in a plate-reading luminometer (GloMax® 96 Microplate, Promega). The activity of caspase 3 was expressed as relative luminescence unit (RLU) with respect to that of mock-infected cells.

### Microarray gene expression analysis of CHIKV-infected HSMM cells

A total of 5 × 10^5^ HSMM cells were seeded in 6-well tissue culture trays (Nunc) and infected in triplicates with CHIKV strain Singapore 072008 at an MOI of 10 or mock infected with an equal volume of concentrated conditioned growth medium. At 6, 12, 24, and 48 h.p.i., total RNA was extracted using TRIzol reagent according to manufacturer’s instructions (Invitrogen). TRIzol lysates were subsequently purified with the RNeasy Mini Kit (Qiagen) and quality tested using the Agilent RNA 6000 Nano Kit on an Agilent bioanalyzer (Agilent Technologies Inc., Germany). The RNA samples were then used for probe synthesis of streptavidin-Cy3-labeled cRNA using the Illumina® TotalPrep RNA Amplification Kit (Illumina Inc., USA) prior to hybridization onto Illumina HumanHT-12 v4 Expression BeadChip gene expression microarray chips (Illumina), which targets 47,231 transcripts derived from the National Center for Biotechnology Information Reference Sequence (NCBI) RefSeq Release 38 (November 7, 2009) and legacy UniGene content. Hybridization was carried out at 65 °C for 18 hrs in an Illumina hybridization oven at 10 rpm. After hybridization, the microarray chips were washed before scanning on Illumina BeadArray Reader. Raw signal data was extracted from the TIFF images with GenomeStudio Data Analysis Software (Gene Expression Module; Illumina).

Raw microarray expression data were processed and analyzed using Partek Genomics Suite (Partek; U.S.A.) to generate values representing fold changes in gene expression. An average of the triplicate values was used to calculate fold change, and each value was then assessed for its statistical significance, using analysis of variance (ANOVA). Host genes demonstrating at least a 2-fold change in expression and a > 95% probability of being expressed differentially (*P* < 0.05) were selected for further investigation. Pathway analysis was subsequently detailed with Ingenuity Pathway Analysis (IPA) 9.0 (Ingenuity Systems 2011, U.S.A.) and differentially regulated genes involved in various pathways were selected for pathway mapping.

### qRT-PCR quantification of CHIKV viral RNA

Viral RNA was extracted using QIAamp Viral RNA Mini Kit (Qiagen), according to manufacturer’s instructions. One-step quantitative real-time reverse transcription-PCR (qRT rt-PCR) assays were performed using the ABI PRISM 7500 RT-PCR system (Applied Biosystems, Life Technologies Corporation, USA). Twenty-five-microliter reaction mixtures were set up according to manufacturer’s instructions, as previously performed in^28^. Briefly, samples were assayed with a final concentration of 250 nM of each CHIKV nsP2 primer in a 1× final concentration of SYBR Green *Taq* Ready Mix for Quantitative RT-PCR (1× *Taq* DNA polymerase, 10 mM Tris-HCl, 50 mM KCl, 3 mM MgCl_2_, 0.2 mM dNTPs, stabilizers) and 1× reference dye (Sigma Aldrich, USA), and Moloney Murine Leukemia Virus reverse transcriptase (M-MLV RT) (Promega). Reaction conditions consist of a 20-min reverse transcription step at 44 °C, followed by 2 min of *Taq* polymerase activation at 94 °C, 40 cycles of PCR at 94 °C for 15 secs per cycle (denaturation) and finally 1 min at 60 °C (annealing and extension). Following the amplification, a melting curve analysis was performed to verify the melting temperature of PCR products amplified by the primer pairs. Amplification graphs were checked for the Ct value of the PCR product. The Ct value represented the cycle by which the fluorescence of a sample increased to a level higher than the background fluorescence in the amplification cycle. β-actin acts as the normalizing housekeeping gene.

### qRT-PCR quantification of host gene expression

Total RNA was extracted using Qiagen RNeasy Mini Kit and treated with DNase (Ambion Turbo DNase), according to manufacturer’s instructions. One-step quantitative real-time reverse transcription-PCR (qRT rt-PCR) assays were performed using the ABI PRISM 7500 RT-PCR system (Applied Biosystems, Life Technologies Corporation, U.S.A.), with similar reaction conditions as previously mentioned. Samples were assayed with a final concentration of 200 nM of each gene primer in a 1× final concentration of Maxima^TM^ SYBR Green/ROX qPCR Master Mix (Hot Start *Taq* DNA polymerase, KCl, (NH_4_)_2_SO_4_, dNTPs, stabilizers) (Fermentas, Thermo Fisher Scientific Inc., U.S.A.) and M-MLV RT (Promega). The comparative threshold cycle (*CT*) method was used to calculate the fold change in gene expression, with β-actin as the normalizing control gene.

### Statistical analysis of results

Calculations of statistical significance were performed using a one-tailed unpaired *t* test in GraphPad Prism software. Data points with *p* values of less than 0.05 were considered statistically significant.

## Additional Information

**How to cite this article**: Hussain, K. M. *et al.* Establishment of a Novel Primary Human Skeletal Myoblast Cellular Model for Chikungunya Virus Infection and Pathogenesis. *Sci. Rep.* **6**, 21406; doi: 10.1038/srep21406 (2016).

## Supplementary Material

Supplementary Information
