# Supplementary material for: Establishment of a Novel Primary Human Skeletal Myoblast Cellular Model for Chikungunya Virus Infection and Pathogenesis
Source: Sci Rep. 2016 Feb 19;6:21406. doi: 10.1038/srep21406 (PMC4759813; doi:10.1038/srep21406)
Supplement: Supplementary Information [file srep21406-s1.pdf]

Supplementary Information

Establishment of a Novel Primary Human Skeletal Myoblast Cellular Model for Chikungunya Virus Infection and Pathogenesis.

Khairunnisa’ Mohamed Hussain, Regina Ching Hua Lee, Mary Mah-Lee Ng and Justin Jang Hann Chu

Suppl. Fig S1

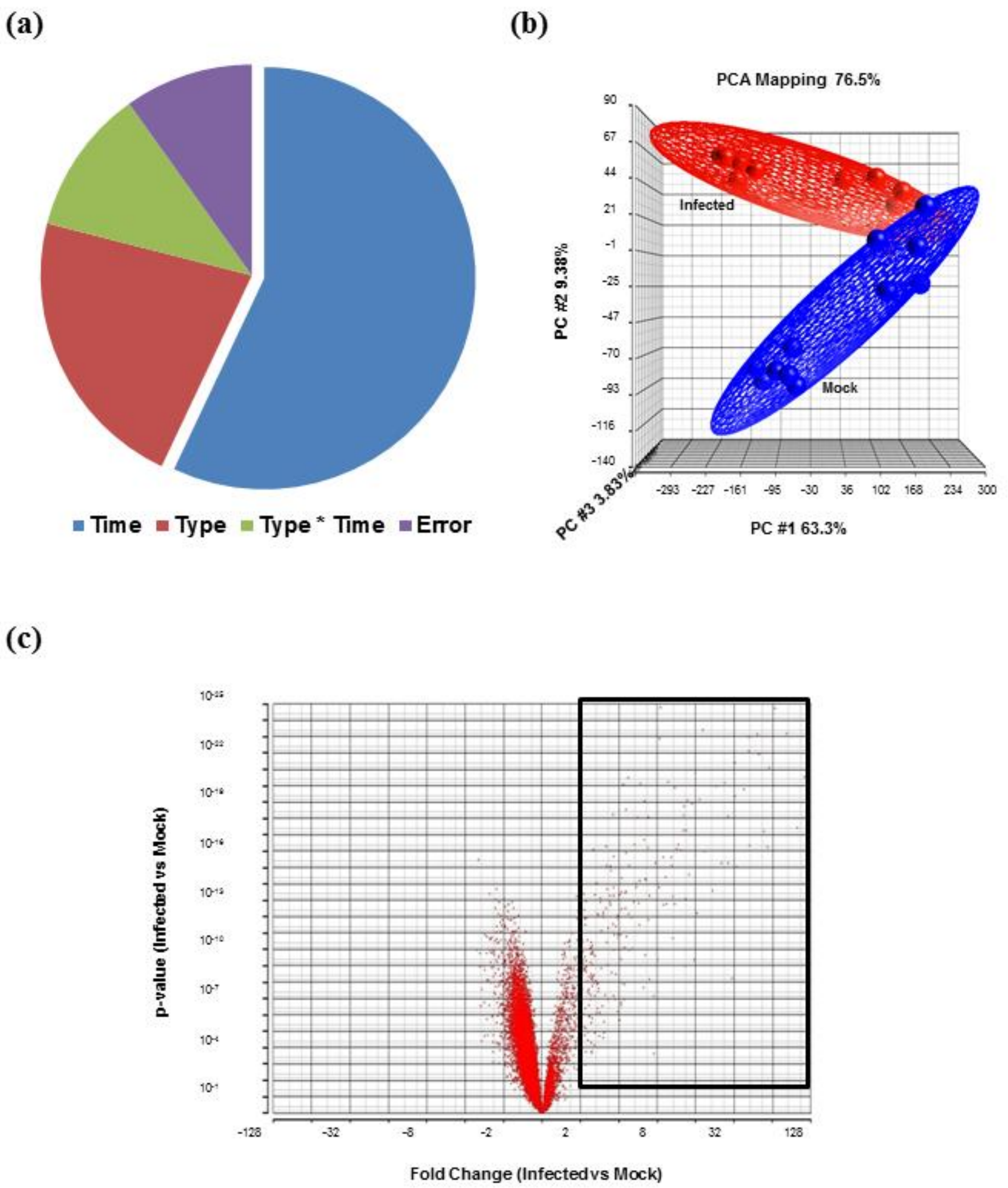

Suppl. Fig S1. Comparison analyses of CHIKV infection on HSMM cells (a) Pie chart displayed the various sources of variation amongst the samples, and infection time-point was found to primarily result in the sample variation (b) PCA plot showing distinct separation between mock (blue) and CHIKV-infected (red) samples, with infection time-point, infection type and a combination of both factors accounting for 76.5% total variation.(c) Volcano plot showing selection criteria for DEGs, with absolute fold changes of more than 2 and p value of less than 0.05 (boxed).

Suppl. Fig S2

Skeletal & Muscular Disorders

Gene	Infected vs Mock	I vs M @ 6 h.p.i.	I vs M @ 12 h.p.i.	I vs M @ 24 h.p.i.	I vs M @ 48 h.p.i.
CXCL10	100.545	241.449	49.986	72.078	117.478
CCL5	54.544	54.680	28.230	45.558	125.855
IFNB1	42.011	94.909	35.549	43.371	21.286
IFIH1	38.450	59.353	28.423	28.766	45.041
TNFSF10	33.405	66.738	15.666	21.148	56.315
TNFSF13B	17.168	26.215	12.794	16.101	16.086
CFB	15.021	12.501	12.248	23.490	14.156
RARRES3	14.224	26.602	7.403	10.677	19.467
GBP1 (inc EG:2633)	12.281	22.813	10.286	9.847	9.844
PSMB9	9.716	9.606	7.809	9.305	12.765
SAMD9L	8.972	15.715	5.609	5.400	13.616
TAP1	8.491	8.444	7.992	9.352	8.238
CXCL9	7.498	42.278	5.286	4.396	3.217
IL6	6.939	27.400	2.847	4.435	6.703
CASP1	6.818	9.535	3.946	4.503	12.753
SP110	6.407	8.625	4.569	4.557	9.381
IL18BP	5.771	6.088	3.493	4.217	12.367
GCA	5.441	3.505	4.823	6.113	8.481
STAT1	5.123	7.163	2.810	3.238	10.569
TLR3	4.826	5.244	3.908	3.879	6.821
CCL3	4.805	10.748	2.873	5.426	3.180
UBE2L6	4.718	4.001	4.659	4.994	5.322
TRIM21	4.539	5.440	3.947	3.688	5.361
CCL3L1	4.479	9.013	2.848	5.524	2.838
IL28A	4.428	13.767	2.859	3.817	2.559
PSMB8	4.349	4.274	3.847	4.210	5.171
CD83	4.112	2.931	3.798	8.323	3.086
NT5C3	3.668	2.585	2.908	3.744	6.430
GBP2	3.632	5.296	3.544	3.778	2.455
HLA-DOB	3.280	6.803	2.148	2.440	3.245
TAP2	3.181	3.685	2.615	3.523	3.016
TNFAIP3	3.003	3.256	2.269	4.318	2.549
BTC	2.914	3.832	2.199	2.264	3.780
CCDC109B	2.700	3.481	2.312	2.262	2.918
CXCL16	2.684	2.722	2.204	2.514	3.441
TRIM69	2.514	2.378	2.634	2.724	2.343
RALGDS	-2.289	-2.487	-2.051	-2.163	-2.488
TNFRSF10D	-2.794	-2.877	-2.853	-3.714	-2.000

Suppl. Fig S2. The list of skeletal and muscular disorders associated host genes that are differentially regulated during CHIKV infection across the different time points of infections.
